# Association between the oxidative balance score and thyroid function: Results from the NHANES 2007–2012 and Mendelian randomization study

**DOI:** 10.1371/journal.pone.0298860

**Published:** 2024-03-18

**Authors:** Liying Song, Haonan Zhou, Qian Yang, Ningyu He, Feifan Fu, Weichao Li, Guosheng Duan, Di Wu, Shuai Hao, Jiaxing Wang, Jing Liu

**Affiliations:** 1 Department of Thyroid Surgery, First Hospital of Shanxi Medical University, Taiyuan, China; 2 Department of Vascular Surgery, Shanxi Bethune Hospital, Shanxi Academy of Medical Sciences and Tongji Shanxi Hospital, Tongji Medical College of HUST, Third Hospital of Shanxi Medical University, Taiyuan, China; 3 First School of Clinical Medicine, Shanxi Medical University, Taiyuan, China; 4 Radiotherapy Department, Shanxi Provincial Peoples Hospital: Fifth Hospital of Shanxi Medical University, Taiyuan, China; 5 Fifth School of Clinical Medicine, Shanxi Medical University, Taiyuan, China; 6 School of Basic Medicine, Shanxi Medical University, Taiyuan, China; 7 School of Management, Shanxi Medical University, Taiyuan, China; King Abdulaziz University Faculty of Medicine, SAUDI ARABIA

## Abstract

**Background:**

Oxidative stress is a significant contributor to the development of various diseases, and the oxidative balance score (OBS) is a valuable tool for assessing the impact of dietary and lifestyle factors on oxidative stress in humans. Nevertheless, the precise relationship between OBS and thyroid function in adults remains elusive.

**Methods:**

This cross-sectional study comprised 6222 adult participants drawn from the National Health and Nutrition Examination Survey (NHANES) conducted from 2007 to 2012. Employing weighted multivariable linear regression modeling, the study estimated the connection between OBS quartiles and thyroid functions. The causal relationship between OBS components and thyroid function was analyzed by Mendelian randomization (MR).

**Results:**

We found a significant negative correlation between OBS and free thyroxine (FT4) and total thyroxine (TT4). Univariate and multivariate MR Analyses showed a causal relationship between BMI and FT4. Copper, smoking, and riboflavin showed a causal relationship with FT4 after moderation.

**Conclusion:**

We found that a lifestyle high in antioxidant exposure reduced FT4 and TT4 levels in the population. We suggest that BMI, Copper, and Riboflavin are important factors in the regulation of FT4 levels.

## 1. Introduction

The physiological functions of the thyroid gland include promoting the metabolism of major nutrients, regulating growth and development, increasing tissue oxygen consumption, promoting energy metabolism, increasing thermogenesis, and improving basal metabolism [1]. Thyroid dysfunction is a common clinical disorder that affects approximately 10% of adults in the general population, and it can affect various body systems and lead to the development of other diseases such as atrial fibrillation, coronary artery disease, and stroke [2–4]. However, even fluctuations in thyroid hormones within the clinically recommended range can also be associated with these pathological conditions. Research has demonstrated that elevations in free Thyroxine (FT4) levels, even within the normal range, can lead to heightened vascular resistance, augmented cardiac contractility, elevated heart rate, and increased left ventricular mass. Such increases can potentially raise the risk of premature atrial contractions, thereby becoming a significant risk factor for atrial fibrillation [5]. Conversely, subclinical hypothyroidism (defined by high thyroid-stimulating hormone (TSH) and FT4 within the reference range) can reduce metabolic rate, increase systemic vascular resistance, promote atherosclerosis, and cause changes in endothelial and coagulation functions [6]. Additionally, among young stroke patients with normal thyroid function, heightened levels of anti-thyroperoxidase antibody (TPOAb) were found to be independently associated with intracranial large artery stenosis [7].

Oxidative stress (OS) results in an excessive accumulation of reactive oxygen species (ROS) that can damage the physiological structure of lipids, proteins, DNA, and RNA, affecting the physiological function of tissues and organs [8]. Studies have shown that oxidative stress is implicated in the pathogenesis of several autoimmune disorders, including thyroid diseases. [9, 10]. A diet high in antioxidants, such as vitamins C, D, and E, can effectively prevent OS while smoking, and alcohol consumption, and a high-fat, high-iron intake diet can promote OS, accelerating ROS accumulation and cellular damage [11].

However, when evaluating the effects of individual antioxidants or pro-oxidants on thyroid function, it is simple to ignore the interactions of multiple antioxidants and pro-oxidants about each other. The oxidation balance score (OBS) is a comprehensive indicator derived from information on diet and other lifestyle factors [12]. An increase in OBS usually indicates an increase in antioxidant exposure [13]. Several studies have investigated the relationship between OBS and various chronic diseases and found a negative association between OBS and a variety of diseases, including type 2 diabetes [14], cardiovascular disease [15], and various types of cancer [16–20].

Although an imbalance between free radical production and antioxidant defense is associated with thyroid dysfunction, there is currently no association detected between OBS and Thyroid Function. Identifying the association between OBS and thyroid function could provide important data for future patient education and therapeutic strategies.This study aims to evaluate the cross-sectional connection between OBS and thyroid function using data from the National Health and Nutrition Examination Survey (NHANES) from 2007 to 2012 MR was used for further study of the relationship between the two.

## 2. Materials and methods

### 2.1 Study population in NHANES

**Fig 1:**
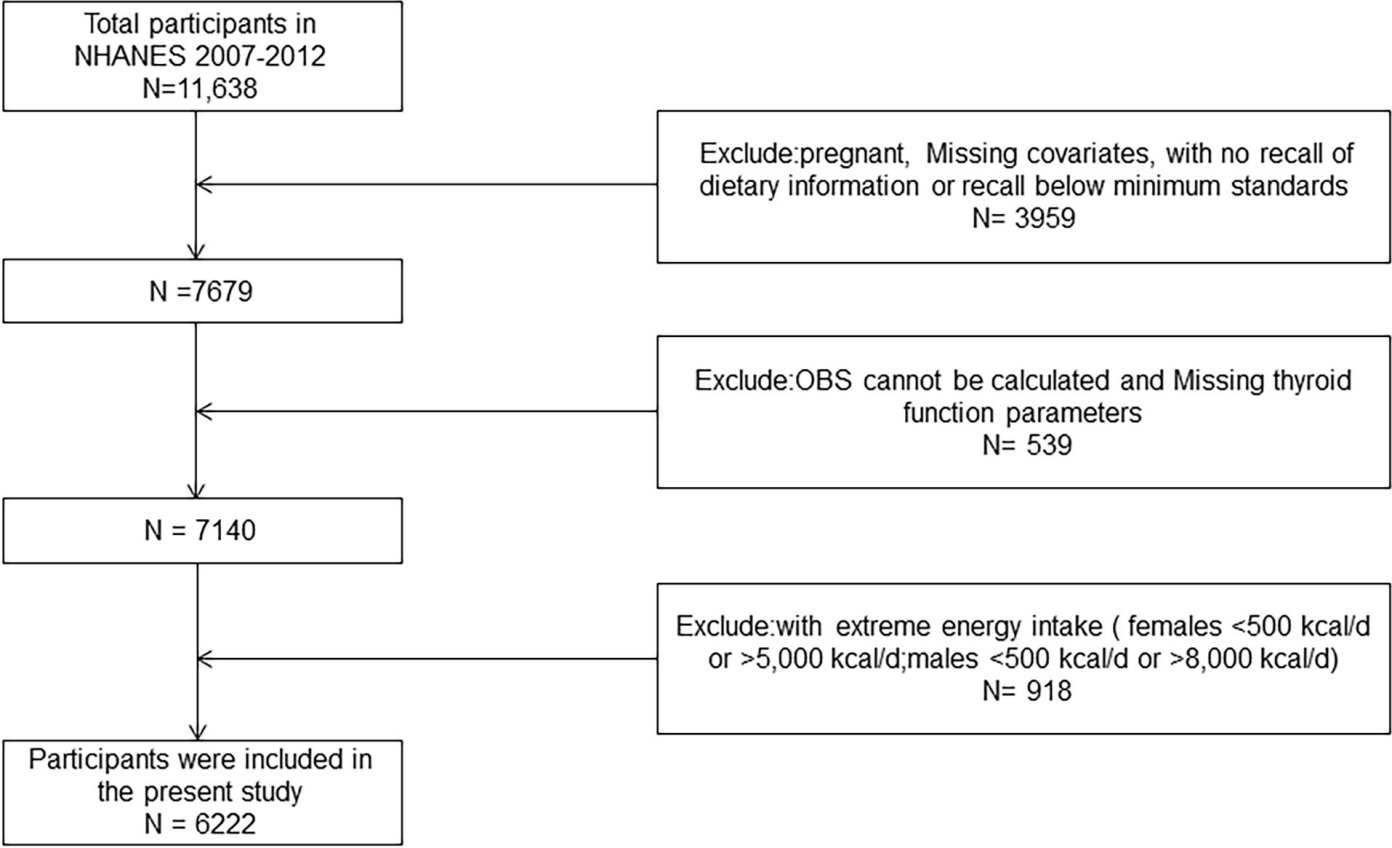
The flowchart depicts the process of screening study participants for the cohort. OBS, oxidative balance score.

The National Health and Nutrition Examination Survey (NHANES) is a comprehensive cross-sectional survey that is managed by the National Center for Health Statistics (NCHS) to assess the health and nutritional status of non-institutionalized American nationals. The NCHS assesses the health and nutritional status of non-institutionalized American nationals by randomly selecting a sample of approximately 5,000 individuals on a two-year cycle through a multistage probability sampling design. The research protocol of NHANES was approved by the National Center for Health Statistics Research Ethics Review Board and written informed consent was obtained. The comprehensive thyroid function data in the NHANES database are available for the years 2007–2012 [21]. In this study, we selected the NHANES survey cycle of 2007 to 2012 and excluded the underage sample (age<18) and the data inclusion exclusion criteria are shown in Fig 1.

### 2.2 Data collection in NHANES

In this research, OBS was employed as an exposure factor. The NHANES 2007–2012 was furnished with dietary consumption data through two 24-hour dietary recall interviews (24HR) conducted at the Mobile Examination Center. The first dietary data were collected personally in the mobile inspection center, and the second interview was conducted by telephone consultation after 3 to 10 days.The construct validity and computation procedures of OBS have been previously documented [11]. Comprising 20 items, the OBS includes both dietary and lifestyle components. The dietary items investigated were Dietary fiber, Carotene, Riboflavin, Niacin, Total folate, Calcium, Magnesium, Zinc, Copper, Selenium, Total fat, Iron, Vitamin B6, B12, C, and E. The OBS lifestyle components encompassed Physical activity, Alcohol, Body mass index, and Cotinine. To calculate the OBS score for each individual, the corresponding score is assigned to each component, and then the scores for each component are summed for each participant. The score allocation scheme for each component is listed in S1 Table. Higher OBS scores were indicative of greater antioxidant potential. In this study, OBS has been deemed a continuous variable and was categorized into quartiles, with the highest quartile (Q4) representing the highest antioxidant potential.

The study’s outcome variable was thyroid function, which was obtained from laboratory data in NHANES 2007–2012. Thyroid parameters, including total and free Thyroxine (T4), total and free Triiodothyronine (T3), thyroglobulin (Tg), thyroglobulin antibodies (TgAb), thyroid peroxidase antibodies (TPOAb), and thyroid-stimulating hormone (TSH), were assessed. The 2007–2012 NHANES Laboratory/Medical Technologists Procedures Manual (LPM) outlines the collection and processing of these thyroid function parameters, and previous studies provide detailed descriptions of sample processing [22]. To account for the influence of iodine intake on thyroid function, UIC was categorized into iodine deficient (<100), normal (100–299), and excessive iodine intake (≥300μg/L) for participants over 18 years old, as determined by a previous study [22].

To control for potential confounding factors, several covariates were included in the analysis. These covariates were age (in years), ethnicity, sex, educational level, poverty income ratio (PIR), smoking status, urine iodine concentration (UIC), and dietary energy intake (in kcal). Ethnicity was stratified into non-Hispanic white, non-Hispanic black, Mexican American, and others. PIR was estimated using poverty guidelines for family size and was categorized as ≤1.3, 1.3–3.5, and >3.5 to reflect the socioeconomic status of participants. Education levels were graded into five groups: less than 9th grade, 9th-11th grade, high school grade/general equivalent diploma, some college or associate degree, and college graduate or above. As recommended by NHANES, smoking status was categorized into ‘Never’, ‘Now’, and ‘Former’. These covariates were included in the regression models as potential confounding variables to adjust for their effects on the association between OBS and thyroid function.

### 2.3 Assumptions and sources of two-sample MR

Most of the GWAS summary data about OBS was obtained from the IEU GWAS database (https://gwas.mrcieu.ac.uk/), while a portion of the GWAS data was sourced from the UK biobank database (http://www.nealelab.is/uk-biobank). We selected only Thyroid Function parameters that exhibited cross-sectional association with OBS as outcome variables and obtained the outcome data from the Thyroidomics Consortium database (https://transfer.sysepi.medizin.uni-greifswald.de/thyroidomics/). Single nucleotide polymorphisms (SNPs) were selected as instrumental variables based on the following criteria: a GWAS-correlated P-value of 5×10−8 or less, an LD r2 of less than 0.001, and location less than 1 megabase (MB) from the index variant. However, if the use of a P-value of 5×10−8 for SNP selection yields a limited number of variants, an alternative strategy may involve using less stringent criteria (P-value of 1×10−5, and F-value > 10) to select SNPs. For IVs that were not present in the outcome, we searched for proxy SNPs in online databases using LD with an r2 greater than 0.8.

### 2.4 Statistical analysis

The statistical analysis in this study was conducted in accordance with CDC guidelines for analyzing complex NHANES data. To account for masked variance, sample weights were assigned to each participant. Continuous variables were reported as mean (SE), while categorical variables were presented as percentages. Multivariable linear regression was used to examine the relationship between OBS and thyroid function, with Model 1 being unadjusted, Model 2 adjusting for age, ethnicity, and sex, and Model 3 being the fully adjusted model. Interaction and stratified analyses were also conducted to explore effect modification by age, sex, and UIC.

For MR analysis, we used the R package "TwoSampleMR". The causal effect was determined using the Wald ratio if one SNP was present in the sample. When the number of SNPs was greater than two, we mostly utilized inverse variance weighting meta-analysis (IVW) for estimating purposes. IVW estimates are the weighted average of the Wald ratios obtained from each SNP. When using the IVW approach, included SNPs cannot have multiple levels of validity to prevent the creation of biased results. MR-Egger is similar to IVW, but it includes an intercept term, allowing for the presence of pleiotropy. Additionally, MR-Egger requires a lower IV concentration than IVW, allowing for the existence of the InSIDE assumption that pleiotropic effects are independent of the correlation between risk variables. The MR-Egger intercept is the average pleiotropic effect of genetic variation, according to the InSIDE hypothesis. Therefore, when pleiotropy was accounted for, MR-Egger estimates were consistent with IVW. The approach of weighted median estimate (WME) can eliminate the effect of erroneous instrumental variables. WME analysis can yield reliable estimates of causal effects even if 50% of the genetic variance information is erroneous. Statistical power analyses were performed with the online mRnd calculator (https://shiny.cnsgenomics.com/mRnd/). The MR Steiger test is used to test the direction of causality in our hypothesis. To detect any outliers and horizontal pleiotropy, we conducted MR Pleiotropy RESidual Sum and Outlier (MRPRESSO), tested directional pleiotropy with MR-Egger intercept, and detected potential heterogeneity with the Cochrane Q test. Radial MR imaging was used to find outliers. Multivariate Mendelian randomization (MVMR) is an extension of conventional univariable MR, which can remove the influence of confounders on causality to obtain a direct effect of exposure on the outcome. Therefore, we performed MVMR to obtain causal estimates adjusted for these genetic correlations.

We utilized R (version 4.1.3) software for all statistical analyses. A p-value of less than 0.05 was considered statistically significant.

## 3. Results

### 3.1 Baseline characteristics

A total of 1,1638 subjects were recruited from the National Health and Nutrition Examination Survey (NHANES) during the year 2007–2012. After applying the inclusion and exclusion criteria, our final sample included 6222 adult participants with an average age and standard error (SE) of 46.58(0.42) and OBS of 21.21(0.28). The sociodemographic weighted characteristics of the study subjects are concisely presented in Table 1 and arranged into quartiles based on their OBS scores. Mean OBS scores ranged from 11.33 (most pro-oxidant) to 29.83 (most anti-oxidant), whereas the four OBS quartile ranges were 4~15, 15~21, 21~26, and 26~36, respectively. Among the four OBS quartiles, for thyroid parameters, differences in TT3 and TT4 were statistically significant. For covariates, except for sex and UIC, the distribution of age, ethnicity, educational level, smoking status, mean dietary energy intake, and PIR were statistically different in 4 groups. Age was similar between the OBS quartile groups. In comparison to OBS Q 1 to 3, individuals in the OBS Q4 group had a greater tendency to be non-Hispanic White (77.98(2.28)). Participants belonging to the OBS Q4 group were observed to exhibit a tendency towards increased energy intake, higher educational attainment, decreased smoking frequency, and higher incomes. According to the analysis, participants belonging to the Q4 group had a statistically significant lower level of TT4 when compared to the other groups. The TT3 mean was recorded as 113.27(0.77) mIU/L, and participants in the Q4 group had higher-than-average TT3 levels. No statistically significant differences were detected among the OBS quartiles in other parameters related to thyroid function (all p-values were greater than 0.05).

**Table 1:** The baseline features of the NHANES (2007–2012) study population in 4 OBS quartile groups.

variable	total	Q1(1720)	Q2(1680)	Q3(1445)	Q4(1377)	Pvalue
age(yrs)	46.58(0.42)	46.89(0.69)	47.58(0.48)	47.06(0.71)	44.94(0.81)	0.01
OBS	21.21(0.28)	11.44(0.11)	18.65(0.07)	23.96(0.06)	29.73(0.10)	< 0.0001
TgAb (IU/mL)	9.46(1.68)	11.39(6.15)	9.89(2.06)	8.41(2.06)	8.31(2.84)	0.9
FreeT3(pg/mL)	3.17(0.01)	3.20(0.02)	3.16(0.02)	3.15(0.02)	3.17(0.02)	0.28
FreeT4 (pmol/L)	10.27(0.07)	10.37(0.11)	10.35(0.08)	10.22(0.08)	10.16(0.11)	0.14
Tg(ug/L)	16.25(0.74)	18.03(1.40)	15.88(1.34)	15.95(0.79)	15.30(2.13)	0.62
TSH(mIU/L)	2.05(0.08)	2.03(0.07)	2.01(0.11)	2.03(0.09)	2.13(0.20)	0.95
TotalT4(μg/dL)	7.87(0.05)	8.09(0.07)	7.98(0.07)	7.75(0.06)	7.66(0.06)	< 0.0001
TotalT3(ng/dL)	113.27(0.77)	115.19(1.33)	112.86(0.88)	111.71(0.98)	113.38(1.30)	0.04
TPOAb(IU/mL)	23.49(1.74)	24.27(5.24)	21.05(3.39)	22.00(2.61)	26.46(4.10)	0.67
energyintakes(kcal)	2147.26(20.16)	1582.84(33.26)	1971.80(26.62)	2271.51(47.03)	2696.23(31.77)	< 0.0001
sex						0.81
Female	52.63(0.02)	51.91(1.59)	51.67(1.71)	53.66(1.50)	53.23(1.75)	
Male	47.37(0.02)	48.09(1.59)	48.33(1.71)	46.34(1.50)	46.77(1.75)	
eth						< 0.0001
White	71.44(0.05)	66.41(3.15)	68.25(3.03)	72.41(2.73)	77.98(2.28)	
Black	10.04(0.01)	15.46(2.10)	11.57(1.75)	7.98(1.03)	5.68(1.01)	
Mexican	7.94(0.01)	7.94(1.28)	9.14(1.21)	8.93(1.40)	5.91(0.94)	
Other	10.59(0.01)	10.19(1.76)	11.03(1.65)	10.68(1.39)	10.44(1.59)	
edu(%)						< 0.0001
Less Than 9th Grade	5.48(0.00)	9.29(1.13)	6.41(0.66)	4.30(0.71)	2.30(0.41)	
9-11th Grade	12.09(0.01)	17.66(1.70)	13.83(1.22)	10.77(1.01)	6.76(0.86)	
High school graduate or equivalent	23.63(0.02)	29.16(1.96)	26.45(2.00)	22.93(1.72)	16.75(1.78)	
Some College or AA degree	29.82(0.02)	26.90(1.79)	31.68(1.50)	30.59(1.54)	29.94(1.68)	
College Graduate or above	28.98(0.02)	16.99(2.02)	21.63(1.84)	31.42(2.02)	44.24(2.38)	
PIR						< 0.0001
≤1.3	21.21(0.01)	31.55(2.36)	20.82(1.67)	18.51(1.83)	14.93(1.69)	
1.3–3.5	34.44(0.02)	36.17(1.80)	40.99(1.99)	32.74(1.76)	28.27(2.28)	
>3.5	44.35(0.03)	32.28(2.59)	38.19(2.52)	48.74(2.54)	56.79(2.65)	
BMI						< 0.0001
<25(normal)	31.35(0.02)	23.65(1.54)	26.54(1.54)	29.90(1.93)	43.98(2.04)	
25–29.9(overweight)	33.44(0.02)	32.11(2.04)	34.81(1.28)	35.31(2.12)	31.60(1.94)	
≥30(obese)	35.21(0.02)	44.23(2.00)	38.65(1.59)	34.79(2.32)	24.42(1.88)	
smoke(%)						< 0.0001
never	54.29(0.03)	43.46(2.20)	51.20(2.28)	57.93(1.83)	63.40(1.86)	
former	24.60(0.01)	20.71(1.37)	26.37(1.61)	25.16(1.69)	25.83(1.76)	
now	21.12(0.01)	35.83(2.35)	22.43(1.52)	16.91(1.28)	10.77(1.33)	
UIC						0.75
<100(iodine deficient)	32.94(0.01)	33.81(1.93)	31.76(2.19)	31.27(1.69)	34.80(2.22)	
100-299(normal)	48.71(0.03)	47.80(2.03)	49.51(2.14)	49.34(1.87)	48.17(2.63)	
≥300(excessive iodine intake)	18.36(0.02)	18.39(1.96)	18.73(1.37)	19.40(1.45)	17.03(1.50)	

For continuous variables, p-value was calculated by weighted t-test. For categorical variables, the p-value was calculated by the weighted chi-square test.

### 3.2 OBS score and thyroid function

We carried out an analysis utilizing multivariable linear regression to explore the correlation between the OBS score and thyroid function, as depicted in Table 2. When the OBS score was considered as a continuous variable, our results showed a statistically significant negative correlation between a higher OBS score and lower FT4 and TT4 levels in crude Model 1. Precisely, each incremental unit increase in the OBS score resulted in a corresponding decrease in the TT4 level by 0.02 ng/dl and the FT4 decreased by 0.01 ng / dl. After grouping the OBS score quartiles, this association was still statistically significant. In model 2, our findings indicated a negative correlation between the quartile range of OBS scores and FT3 levels, which, however, ceased to exist after additional adjustment for confounding factors. Furthermore, there was no statistically significant correlation between OBS and other thyroid function parameters (p > 0.05).

**Table 2:** Association between oxidative balance score and thyroid function among U.S. adult participants in NHANES from 2007 to 2012.

	Variable	Continuous		Quartile 1	Quartile 2		Quartile 3		Quartile 4	
		β(95% CI)	p-value		β(95% CI)	p-value	β(95% CI)	p-value	β(95% CI)	p-value
Model 1	TgAb (IU/mL)	-0.09 (-0.41,0.24)	0.60	ref	5.3 (-0.83,11.42)	0.09	0.43 (-5.94, 6.81)	0.89	-0.39 (-6.85, 6.07)	0.91
	Free T3 (pg/mL)	0 (0.00,0.00)	0.57	ref	-0.01 (-0.05,0.02)	0.51	0.01 (-0.02,0.05)	0.52	-0.01 (-0.04,0.03)	0.77
	Free T4 (pmol/L)	-0.01 (-0.02,0.00)	**0.02**	ref	-0.04 (-0.18, 0.09)	0.53	-0.16 (-0.30, -0.01)	**0.03**	-0.14 (-0.29, 0.00)	**0.05**
	Tg (ug/L)	-0.22 (-0.39, -0.05)	**0.01**	ref	-1.43 (-4.60, 1.74)	0.38	-2.25 (-5.55, 1.05)	0.18	-4.66 (-8.00, -1.32)	**0.01**
	TSH (mIU/L)	0 (-0.01,0.01)	0.80	ref	0.03 (-0.16,0.23)	0.74	-0.03 (-0.23,0.18)	0.78	0 (-0.21,0.21)	0.98
	Total T4 (μg/dL)	-0.02 (-0.03, -0.01)	**<0.0001**	ref	-0.05 (-0.16, 0.05)	0.33	-0.24 (-0.36, -0.13)	**<0.0001**	-0.36 (-0.47, -0.24)	**<0.0001**
	Total T3 (ng/dL)	-0.04 (-0.13,0.04)	0.32	ref	0.12 (-1.52,1.76)	0.89	-0.38 (-2.09,1.33)	0.67	-0.06 (-1.79,1.67)	0.94
	TPOAb (IU/mL)	-0.02 (-0.36,0.32)	0.89	ref	0.19 (-6.29,6.67)	0.95	0.01 (-6.73,6.75)	1.00	-1.01 (-7.84,5.83)	0.77
Model 2	TgAb (IU/mL)	-0.11 (-0.43, 0.22)	0.52	ref	4.96 (-1.16,11.09)	0.11	0.14 (-6.25, 6.54)	0.96	-0.59 (-7.12, 5.94)	0.86
	Free T3 (pg/mL)	0 (0.00, 0.00)	**0.004**	ref	-0.02 (-0.05, 0.01)	0.23	-0.01 (-0.05, 0.02)	0.53	-0.05 (-0.08, -0.01)	**0.01**
	Free T4 (pmol/L)	-0.01 (-0.02, 0.00)	**0.02**	ref	-0.05 (-0.19, 0.09)	0.48	-0.16 (-0.30, -0.01)	**0.03**	-0.13 (-0.28, 0.01)	0.07
	Tg (ug/L)	-0.14 (-0.31, 0.03)	0.11	ref	-1.02 (-4.18, 2.14)	0.53	-1.24 (-4.55, 2.06)	0.46	-3.14 (-6.51, 0.23)	0.07
	TSH (mIU/L)	0 (-0.01, 0.01)	0.55	ref	0.01 (-0.18, 0.21)	0.89	-0.06 (-0.26, 0.15)	0.57	-0.03 (-0.24, 0.18)	0.81
	Total T4 (μg/dL)	-0.02 (-0.02, -0.01)	**<0.0001**	ref	-0.06 (-0.17, 0.04)	0.24	-0.24 (-0.35, -0.12)	**<0.0001**	-0.32 (-0.44, -0.20)	**<0.0001**
	Total T3 (ng/dL)	-0.13 (-0.21, -0.04)	**0.03**	ref	-0.33 (-1.91, 1.25)	0.68	-1.34 (-2.99, 0.31)	0.11	-1.67 (-3.36, 0.01)	**0.05**
	TPOAb (IU/mL)	-0.07 (-0.42, 0.27)	0.68	ref	-0.76 (-7.22, 5.71)	0.82	-1.02 (-7.77, 5.74)	0.77	-1.68 (-8.57, 5.22)	0.63
Model 3	TgAb (IU/mL)	-0.17 (-0.52, 0.18)	0.34	ref	4.2 (-1.99,10.38)	0.18	-0.65 (-7.20, 5.89)	0.84	-1.56 (-8.39, 5.26)	0.65
	Free T3 (pg/mL)	0 (-0.01, 0.00)	0.22	ref	-0.01 (-0.04, 0.03)	0.71	0.01 (-0.03, 0.04)	0.67	-0.02 (-0.06, 0.02)	0.32
	Free T4 (pmol/L)	-0.01 (-0.02, -0.01)	**<0.001**	ref	-0.08 (-0.22, 0.06)	0.25	-0.22 (-0.36, -0.07)	**0.003**	-0.23 (-0.39, -0.08)	**0.003**
	Tg (ug/L)	-0.06 (-0.24, 0.12)	0.53	ref	-0.44 (-3.64, 2.75)	0.79	-0.26 (-3.64, 3.12)	0.88	-1.76 (-5.29, 1.76)	0.33
	TSH (mIU/L)	0 (-0.02, 0.01)	0.38	ref	0 (-0.20, 0.20)	0.98	-0.08 (-0.29, 0.13)	0.46	-0.05 (-0.27, 0.17)	0.64
	Total T4 (μg/dL)	-0.02 (-0.02, -0.01)	**<0.0001**	ref	-0.06 (-0.17, 0.05)	0.31	-0.22 (-0.34, -0.11)	**<0.0001**	-0.31 (-0.43, -0.19)	**<0.0001**
	Total T3 (ng/dL)	-0.03 (-0.11, 0.06)	0.58	ref	0.49 (-1.10, 2.08)	0.54	-0.05 (-1.74, 1.63)	0.95	0.18 (-1.58, 1.93)	0.84
	TPOAb (IU/mL)	-0.22 (-0.58, 0.15)	0.25	ref	-2 (-8.53, 4.53)	0.55	-2.97 (-9.88, 3.94)	0.40	-4.21 (-11.41, 3.00)	0.25

Model 1: unadjusted; Model 2: age, race, and sex were adjusted; Model 3: age, race, smoking status, education level, UIC, and PIR were adjusted.

We stratified our analysis by age, sex, and UIC to account for the effects of age, sex, and iodine intake on thyroid function.We found a significant negative association between TT4 and FT4 with OBS scores in both female and male groups. Additionally, a strong negative relationship between OBS and TSH and TPOAb was identified in the male group. Specifically, TPOAb decreased by 0.74 IU/mL for every unit increase in OBS; participants in Q4 exhibited a mean decrease of 0.18 mIU/L in TSH compared with those in Q1 (S2 Table). In the excessive iodine intake group, the negative correlation between FT4 and OBS scores disappeared. Unexpectedly, in the normal iodine intake group, a more apparent negative correlation was observed between TT4 and OBS (p for interaction = 0.003) (S3 Table). We categorized age into two groups: <45 years and ≥45 years, and observed a significant negative correlation between both TT4 and FT4 levels and OBS scores. This negative correlation was notably stronger in the age group <45 (p for interaction = 0.044) (Table 3). Furthermore, in the age group ≥45, we noted a significant positive correlation between the level of TT3 and OBS, with statistically significant between-group heterogeneity (p for interaction < 0.001).Our findings suggest that higher OBS scores (antioxidant) may lead to lower free and total T4 levels. After subgroup analysis, the negative association between OBS and total and free T4 appeared to be stronger in the age group <45. We also observed a significant negative correlation between TSH and TPOAb levels and OBS in the male groups, as well as a significant negative correlation between TT4 in the normal iodine intake group and OBS scores.

**Table 3:** The table of the relationship between OBS and thyroid function within age subgroups.

Age	Variable	Continuous		Quartile 1	Quartile 2		Quartile 3		Quartile 4	
		β(95% CI)	p-value		β(95% CI)	p-value	β(95% CI)	p-value	β(95% CI)	p-value
<45	TgAb (IU/mL)	-0.25 (-0.57, 0.08)	0.14	ref	-4.19 (-10.15, 1.78)	0.17	-2.53 (-8.65, 3.59)	0.42	-6.62 (-12.96, -0.27)	**0.04**
	Free T3 (pg/mL)	0 (-0.01, 0.00)	0.50	ref	-0.01 (-0.08, 0.06)	0.81	0.01 (-0.06, 0.09)	0.71	-0.02 (-0.09, 0.06)	0.68
	Free T4 (pmol/L)	-0.02 (-0.03, -0.01)	**<0.001**	ref	-0.14 (-0.33, 0.06)	0.16	-0.4 (-0.60, -0.20)	**<0.001**	-0.33 (-0.53, -0.12)	**0.002**
	Tg (ug/L)	-0.04 (-0.18, 0.09)	0.55	ref	-0.97 (-3.47, 1.53)	0.45	0.58 (-1.98, 3.15)	0.66	-1.22 (-3.88, 1.44)	0.37
	TSH (mIU/L)	-0.01 (-0.03, 0.00)	0.12	ref	-0.08 (-0.39, 0.22)	0.59	-0.12 (-0.43, 0.19)	0.46	-0.3 (-0.62, 0.03)	0.07
	Total T4 (μg/dL)	-0.02 (-0.03, -0.01)	**<0.0001**	ref	0.04 (-0.13, 0.21)	0.66	-0.26 (-0.43, -0.09)	**0.003**	-0.29 (-0.47, -0.11)	**0.002**
	Total T3 (ng/dL)	-0.12 (-0.26, 0.02)	0.08	ref	-1.13 (-3.66, 1.40)	0.38	-2.77 (-5.37, -0.17)	**0.04**	-0.9 (-3.60, 1.79)	0.51
	TPOAb (IU/mL)	-0.23 (-0.73, 0.27)	0.36	ref	-1.03 (-10.22, 8.16)	0.83	-0.15 (-9.58, 9.28)	0.98	-6.72 (-16.50, 3.06)	0.18
≥45	TgAb (IU/mL)	-0.18 (-0.73, 0.37)	0.52	ref	8.71 (-0.71,18.12)	0.07	-0.46 (-10.67, 9.74)	0.93	0.27 (-10.55,11.09)	0.96
	Free T3 (pg/mL)	0 (0.00, 0.00)	0.21	ref	0 (-0.03, 0.04)	0.86	0.02 (-0.02, 0.05)	0.31	0.02 (-0.01, 0.06)	0.21
	Free T4 (pmol/L)	-0.02 (-0.03, -0.01)	**<0.001**	ref	-0.09 (-0.28, 0.10)	0.34	-0.16 (-0.37, 0.04)	0.12	-0.33 (-0.55, -0.12)	**0.003**
	Tg (ug/L)	-0.04 (-0.33, 0.25)	0.81	ref	0.1 (-4.89, 5.09)	0.97	-0.48 (-5.89, 4.93)	0.86	-1.72 (-7.46, 4.02)	0.56
	TSH (mIU/L)	0 (-0.02, 0.01)	0.87	ref	0.03 (-0.23, 0.29)	0.83	-0.08 (-0.37, 0.20)	0.57	0.09 (-0.21, 0.40)	0.55
	Total T4 (μg/dL)	-0.01 (-0.02, -0.01)	**<0.001**	ref	-0.1 (-0.25, 0.04)	0.15	-0.16 (-0.31, -0.01)	**0.04**	-0.26 (-0.43, -0.10)	**0.002**
	Total T3 (ng/dL)	0.19 (0.07, 0.31)	**0.002**	ref	2.04 (-0.03, 4.12)	0.05	2.95 (0.70, 5.20)	**0.01**	3.57 (1.19, 5.96)	**0.003**
	TPOAb (IU/mL)	-0.22 (-0.74, 0.30)	0.40	ref	-2.63 (-11.59, 6.34)	0.57	-4.98 (-14.70, 4.74)	0.32	-2.53 (-12.84, 7.78)	0.63

In the subgroup analysis, all covariates except age were adjusted.

### 3.3 The results of MR analysis

**Fig 2:**
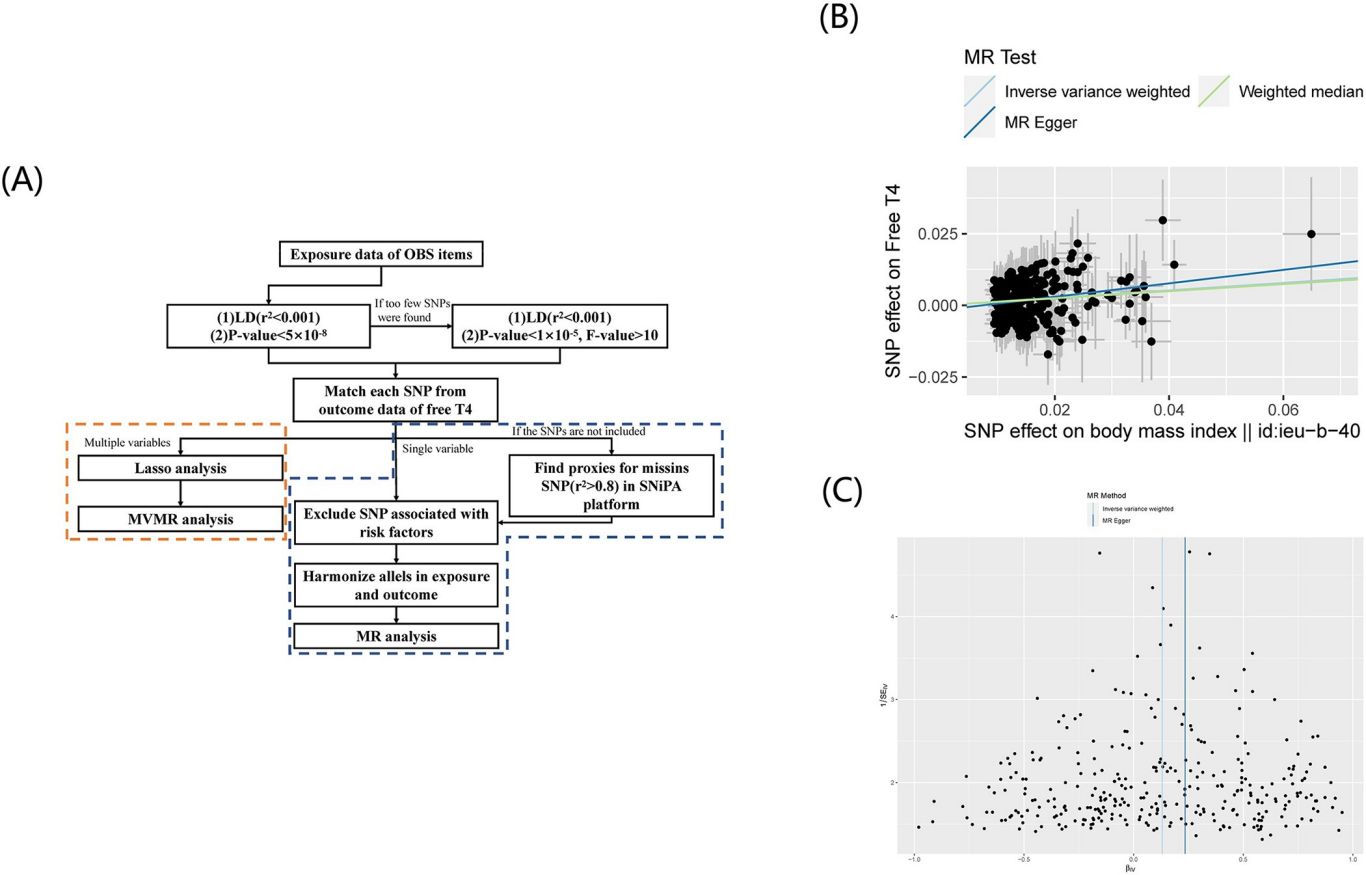
(a) The flow chart describing the methods and basic flow of MR analysis applied in this study. (b) Scatter plots for MR analyses of the causal effect of BMI on Free T4. (c) Funnel plot of MR analyses of the causal effect of BMI on Free T4.

After conducting a correlation analysis of the cross-sectional study, we performed an MR analysis of the parameters of Thyroid Function associated with OBS. The figure illustrates the screening process of instrumental variables (IVs) and the flow chart of MR analysis (Fig 2A). As public GWAS data for TT4, dietary fiber, niacin, and total folate have not been published, we only carried out MR analysis for the remaining OBS items and FT4. Table 4 presents detailed information on included single-nucleotide polymorphisms (SNPs).

**Table 4:** The table of the information on GWAS summary data for 16 OBS items.

Outcome traits	Region	Publish year	N of SNPs	Sample size	Source	Strength		Cochran’s Q P-value	A p value of Egger_intercept	P value of MRRRSSO global test
						R^2^	Total F value			
Carotene	Europe	2018	19	64979	ukb-b-16202	0.006	21.49	0.250	0.313	0.287
Riboflavin	Europe	2018	6	3301	Prot-a-2529	0.040	23.14	0.217	0.870	0.357
Vitamin B6	Europe	2018	20	64,979	ukb-b-7864	0.007	21.96	0.134	0.103	0.051
Vitamin B12	Europe	2018	16	64,979	ukb-b-19524	0.005	20.66	0.067	0.046	0.021
Vitamin C	Europe	2018	19	64,979	ukb-b-19390	0.006	21.64	0.116	0.077	0.056
Vitamin E	Europe	2018	26	64,979	ukb-b-6888	0.008	21.27	0.041	0.673	0.051
Calcium	Europe	2018	18	64,979	ukb-b-8951	0.006	21.89	0.339	0.671	0.359
Magnesium	Europe	2018	23	64979	ukb-b-7372	0.007	20.99	0.150	0.112	0.118
Zinc	Europe	2018	3	461384	ukb-b-13891	0.000	1.26	0.028	0.701	NA
Copper	Europe	2013	2	2603	ieu-a-1073	0.040	53.98	0.866	NA	NA
Selenium	Europe	2013	12	2874	ieu-a-1075	0.110	29.53	0.096	0.932	0.152
Total fat	Europe	2018	18	64979	ukb-b-12379	0.006	21.29	0.070	0.236	0.069
Iron	Europe	2018	15	64979	ukb-b-20447	0.005	20.56	0.100	0.289	0.104
Alcohol	Europe	2018	27	64,979	ukb-b-5359	0.006	15.12	0.029	0.901	0.041
Body mass index	Europe	2018	312	681,275	ieu-b-40	0.026	67.39	1.000	0.205	1.000
Smoking	Europe	2019	208	607,291	ieu-b-4877	0.039	124.20	0.003	0.153	0.735

CI: confidence interval; SNP: single nucleotide polymorphism; MR-PRESSO: Mendelian randomization pleiotropy residual sum and outlier.

**Fig 3:**
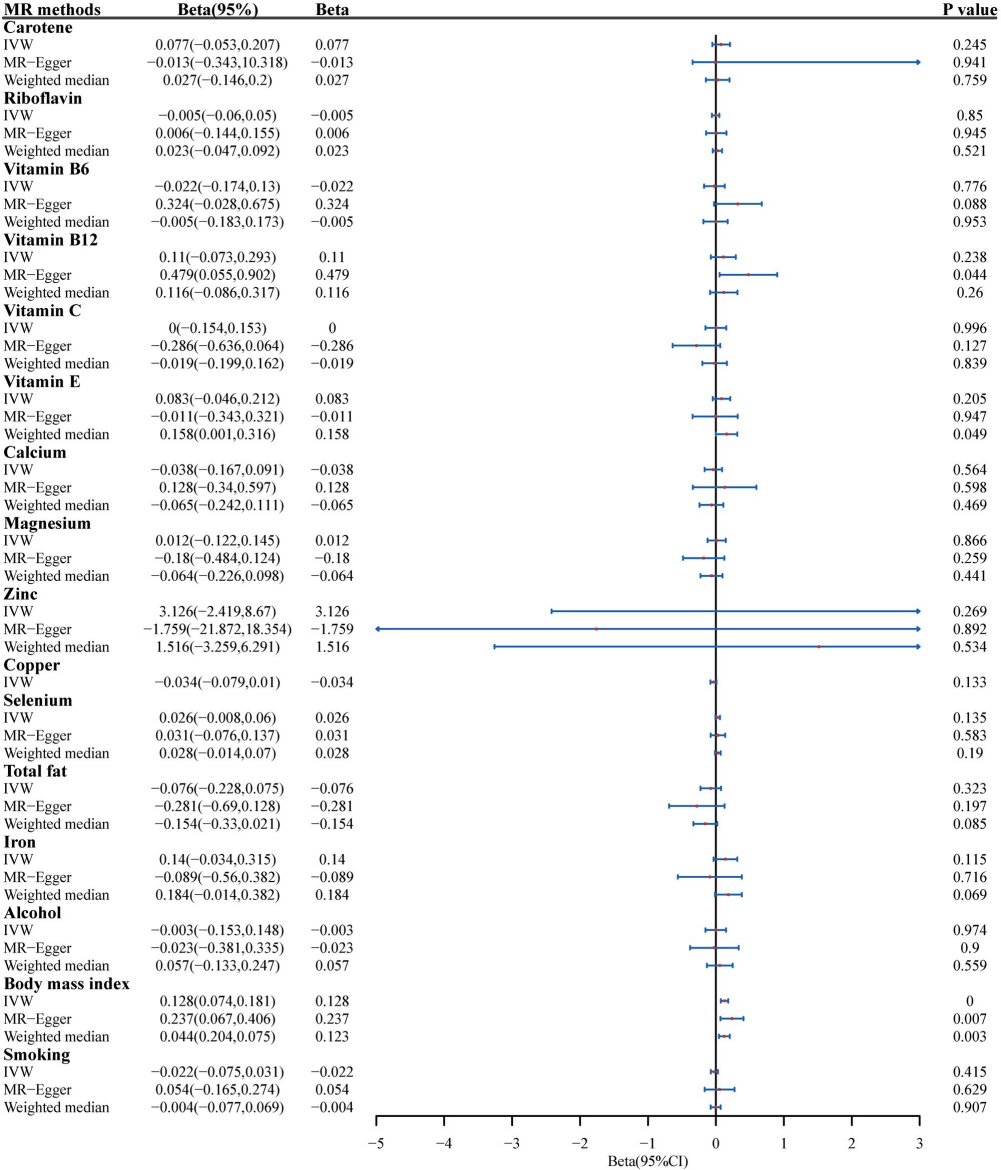
Forest plot of Univariate Mendelian randomization results of each 16 OBS items with Free T4.

According to the IVW analysis results, we unexpectedly found a significant causal relationship of BMI with FT4 (β = 0.13, 95% CI = 0.07 ~ 0.18, P < 0.001) (Fig 2B) and no horizontal pleiotropy (intercept of MR-Egger regression = −0.0017, P = 0.204), but there was significant heterogeneity, and MRPRESSO suggested outliers. After excluding the outlier reported by radial MR and MRPRESSO, this result no longer shows heterogeneity (Q statistics = 310, P = 1) (Fig 2C), and no SNP was observed in the leave-one-out plot (S1 Fig) that could significantly affect the overall results. This result removed rs10747488, rs11496125, rs12299814, rs12328930, rs12593036, rs12981256, rs13021737, rs1421334, rs1477199, rs17056301, rs17405819 rs17724992, rs2065418, rs273504, rs2820311, rs331966, rs4072917, rs4639527, rs4757144, rs6985109, rs7334078, rs769449, and rs784944 (S4 Table). According to the PhenoScanner database these SNPs were considered to be associated with confounding, such as schizophrenia [23], depression [24], and systolic and diastolic blood pressure [25]. MR analysis of the other items did not show a causal relationship with freeT4 (Fig 3). In sensitivity analysis, Cochran’s Q statistic indicated significant heterogeneity among SNPs for Vitamin E, Zinc, and Alcohol. Moreover, the intercept of the MR-Egger regression in Body mass index and Vitamin B6 were significantly different from zero, indicating a horizontal pleiotropy of IV in these items. Therefore, we chose MR-Egger as the method of causal effect evaluation in these items. Unfortunately, the MR-Egger results still failed to reveal any significant causal relationship. It is worth noting that all of these IVs have F-values between 20 and 125 and, thus, are largely unaffected by the bias generated by weak instrumental variables. All results of the MR Steiger directionality test illustrated the accuracy of our estimate of causal direction.

The MVMR results showed that BMI remained significantly causally related to FT4 when we included all 17 OBS items in the analysis. Considering that the large number of included exposures is prone to collinearity, we used LASSO analysis to remove the collinearity exposures, and unfortunately they were all collinearity with each other in different degrees. Therefore, we classified them into pro-oxidant and antioxidant groups for MVMR analysis based on their different oxidation. For the pro-oxidant group, the LASSO analysis left BMI, A, and B. After MVMR analysis, we can observe that BMI remains causally related to FT4 (IVW: β = -0.03, 95% CI = 0.02 ~ 0.16, P  = 0.02) and smoking also has a causal relationship with free T4 (IVW: β = -0.10, 95% CI = -0.20 ~ -0.00, P  = 0.04). The antioxidant group retained Riboflavin, Copper, and C by LASSO analysis, while the results of MVMR were surprising, showing significant causality of Riboflavin (IVW: β = -0.03, 95% CI = -0.10 ~ -0.01, P  = 0.01) and Copper (IVW: β = -0.02, 95% CI = -0.04 ~ -0.00, P  = 0.02) with free T4 by adjustment.

## 4. Discussion

Our study aimed to evaluate the connection between OBS and thyroid function with the aid of a multivariable linear regression model. After dividing the participants into four OBS quartiles, we compared the distribution of the different covariates. The findings revealed that non-Hispanic whites, the wealthy, never smokers, and highly educated people in the quartile group Q1–Q4 received higher OBS scores, indicating a greater exposure advantage for antioxidants in their diets. This may be related to the fact that this segment of the population pays more attention to physical health and maintaining good living habits. In the meantime, we discovered a statistically significant negative correlation between OBS and TT3, and TT4. However, when the model was further adjusted for potential confounders, the correlation between TT3 and OBS was no longer significant but the negative correlations between freeT4, totalT4, and OBS were significantly exposed. Similar conclusions to ours have been reported in previous research, where higher dietary selenium (an antioxidant) intake was associated with a decline in serum T4 concentrations but not with a corresponding change in TSH levels [26]. The potential mechanism is still unclear and needs to be proven by further in vitro and in vivo experiments.

Considering the effects of age, gender, and iodine intake on thyroid function, we performed a subgroup analysis for these three variables. We found that the negative correlation between OBS and TSH and TPOAb levels were more significant in men, which may be related to the protective effect of estrogen against oxidative stress [27, 28]. When subgroup analysis was performed based on UIC, we found that OBS was negatively associated with TT4 and FT4 within normal iodine intake in adults. However, in the groups with high iodine intake, this difference lost statistical significance. Previous studies illustrated that iodine has antioxidant properties and can increase the antioxidant capacity of human serum [29], but the effect of iodine on thyroid function may counteract this effect. In the age subgroup analysis, TT4 and FT4 levels were significantly negatively correlated with OBS in both the young and middle-aged as well as the elderly individuals, with the younger and middle-aged groups (age < 45) showing a greater sensitivity to antioxidant diets. This could be attributed to the heightened glutathione peroxidase activity in older cells, rendering them more resistant to oxidative stress compared to younger cells [30]. In the elderly population, we found a significant positive correlation between TT3 level and OBS score, which may be due to the increase of deiodinase activity and the conversion of T4 to T3 caused by the intake of antioxidants against oxidative stress [31]. At the same time, studies have shown that higher T3 levels are associated with longevity [32]. Several studies have demonstrated that higher serum-FT4 levels are considered a risk factor for the development of cardiovascular disease and death within the range of clinical recommendations [33, 34]. By contrast, relatively low levels of FT4 were conducive to delaying aging by reducing metabolic demand, and normal subjects with lower FT4 levels lived 3.7 years longer on average than normal subjects with higher FT4 levels [35, 36]. Therefore, we recommend augmenting the intake of antioxidants in daily life.

After MR analysis between the 16 entries of OBS and FT4, we further found a significant positive causal relationship between BMI with FT4, and this result remained significant under the adjustment of MVMR, and this is consistent with the results of our cross-sectional study. The results of the cross-sectional study showed that FT4 levels were negatively correlated with OBS, higher antioxidant exposure was associated with lower serum FT4 levels, while BMI as a prooxidant, increased BMI was associated with higher serum FT4 levels. Our results of MR are more clear about their relationship, and the robustness of these results gives us reason for believing that BMI is a key factor influencing FT4 levels. It is worth noting that the relationship between BMI and serum FT4 remains a topic of debate, with some studies reporting a negative correlation between the two variables. This phenomenon can be explained by two factors: Firstly, hypothyroidism is known to decrease the body’s metabolic rate and often coexists with obesity [37]. Secondly, increased leptin levels in obesity can influence the hypothalamus-pituitary-thyroid (HPT) axis and consequently the endocrine system. Studies have shown that leptin levels and TSH levels are positively correlated and that TSH can increase the level of deiodinase, leading to the preferential production of T3 by thyroid cells, resulting in an increase in serum FT3 levels, but not FT4. This may account for the decrease in serum FT4 levels observed in individuals with higher BMI [38–42]. However, another study reported a positive association between BMI and FT4.They showed that morbidly obese subjects had higher levels of TT3, FT3, TT4, and TSH than those of the control group, probably as a result of the reset of their central thermostat at higher levels [43]. In addition,individuals with higher BMI often experience increased oxidative stress in accumulated fat, leading to dysregulation of adipocytokine production and selective increases in ROS, which can result in elevated systemic oxidative stress. Excessive production of ROS can cause hyperthyroidism, often accompanied by low TSH, high FT3, and FT4 [44]. Many articles studying smoking usually use cotinine as a surrogate quantitative measure to illustrate the strong correlation between the two [45, 46]. In the present study, smoking data were used as a proxy because GWAS data for cotinine were not available. After MVMR modulation, smoking, and freeT4 produced a causal effect. Smoking can affect levels of perfluoroalkyl acids (PFAS) in humans, and PFAS is now thought to cause effects on thyroid function, with a positive correlation to FreeT4 levels [47].

Riboflavin and Copper showed significant causal effects after elimination for confounders. Copper has been previously suggested as a microelement for maintaining normal thyroid function, stimulating the synthesis of thyroid hormones, and leading to lower levels of FT4 in the body if it is excessively deficient [48]. Riboflavin, also known as vitamin B2, is a micronutrient that has an important impact on thyroid function, as evidenced by the fact that patients with low vitB2 Patients with lower levels of vitamin B2 usually have lower serum T4 [49]. Our results reveal the causal relationship between them and suggest that they are all key factors influencing freeT4 levels in humans. Therefore, we suggest that normal freeT4 levels can be maintained by improvement of BMI and moderate consumption of Riboflavin and Copper.

This is the first to assess the effects of dietary pro-oxidant and antioxidant protective exposures on thyroid function using a nationally representative U.S. general population. In parallel, we acknowledged that it has some restrictions. (1) Due to poor adherence to the questionnaire among younger individuals, our study only included adults aged ≥ 18. Additionally, pregnant individuals were excluded from the study due to potential dietary abnormalities during pregnancy, so the generalizability of our findings to this population is uncertain (2) Since some of the variables were obtained from the questionnaire, this would lead to an inevitable recall bias in the information we obtained. (3) Due to the lack of clinical research and basic experiments, currently, it is difficult for us to explain the intrinsic mechanism associated with OBS and fluctuations in thyroid hormone levels. (4) Individual-level GWAS data are deficient for further testing of nonlinear causality. (5) The GWAS data used in this study was limited to European populations, and thus, our results may not apply to other populations (6) The study of OBS items with FT4 had a lower p threshold due to a paucity of IVs. While our F-statistic tests revealed no significant risk of weak instrument bias, this negative result should be interpreted with caution.

## 5. Conclusions

In conclusion, our study illustrates that a diet and lifestyle rich in antioxidants is correlated with decreased levels of free and total T4.Therefore, boosting antioxidant intake may serve as a cost-effective measure in preventing cardiovascular and cerebrovascular diseases and in slowing down the aging process, particularly among young and middle-aged individuals. Based on further investigation by MR, we suggest that BMI, Copper, and Riboflavin are important factors affecting freeT4, which can help us to better regulate freeT4 to healthy levels.

## Supporting information

S1 Fig Forest plots of MR leave-one-out sensitivity results (IVW method) for the causal effect of BMI on Free T4. (TIF)

S1 Table Oxidative balance score assignment scheme. (DOCX)

S2 Table The table of the relationship between OBS and thyroid function within gender subgroups. (DOCX)

S3 Table The table of the relationship between OBS and thyroid function within UIC subgroups. (DOCX)

S4 Table Details of the SNPs that were finally included in the MR analysis. (CSV)
