# Supplementary figures and images for: Association between the oxidative balance score and thyroid function: Results from the NHANES 2007–2012 and Mendelian randomization study
Source: PLoS One. 2024 Mar 18;19(3):e0298860. doi: 10.1371/journal.pone.0298860 (PMC10947682; doi:10.1371/journal.pone.0298860)

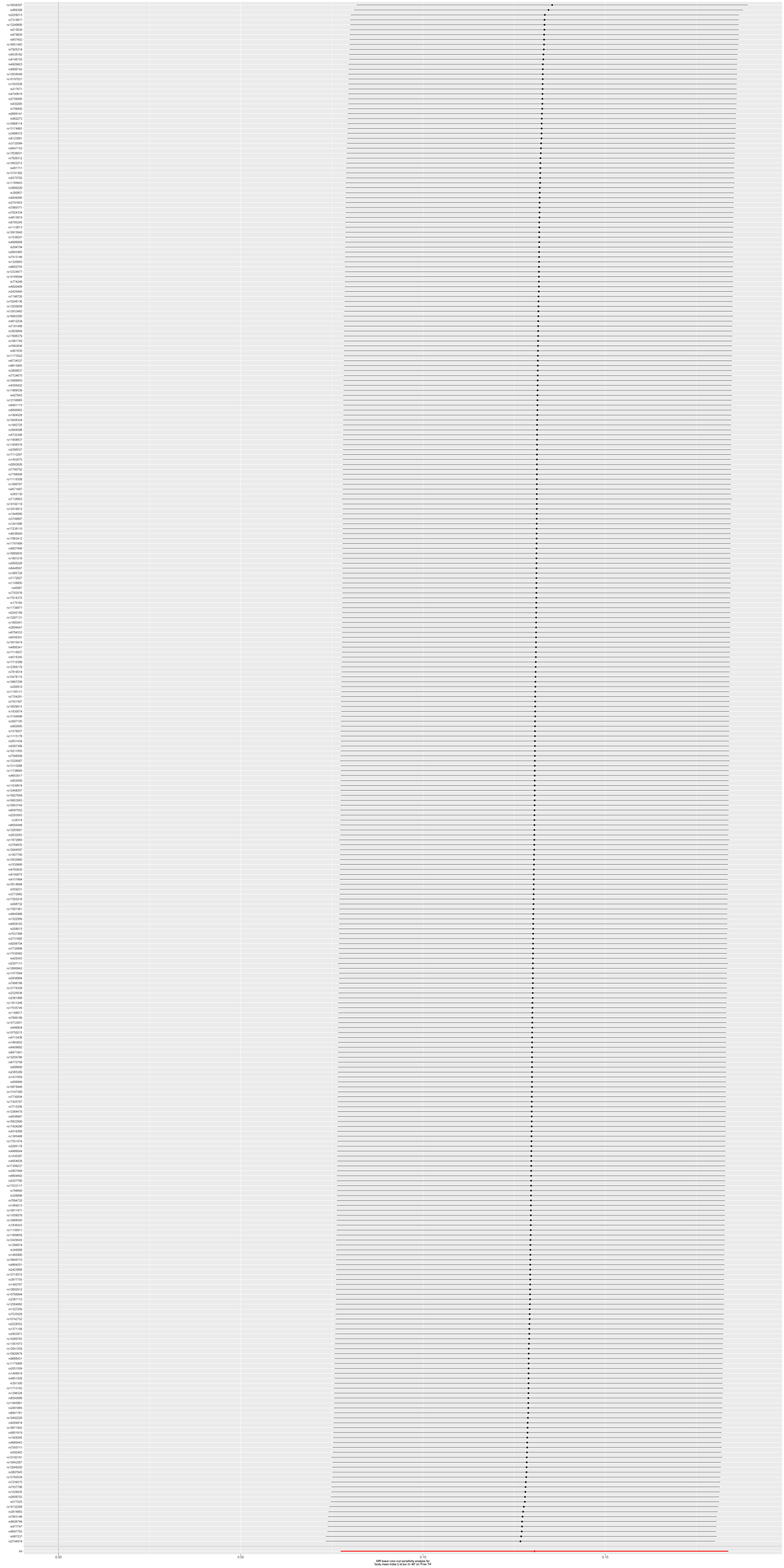

Supplement: S1 Fig — (TIF) [file pone.0298860.s001.tif]
