# Supplementary material for: Association between the oxidative balance score and thyroid function: Results from the NHANES 2007–2012 and Mendelian randomization study
Source: PLoS One. 2024 Mar 18;19(3):e0298860. doi: 10.1371/journal.pone.0298860 (PMC10947682; doi:10.1371/journal.pone.0298860)
Supplement: S2 Table — (DOCX) [file pone.0298860.s003.docx]

**S2 Table. The table of the relationship between OBS and thyroid function within gender subgroups.**

| Gender | Variable | Continuous | | Quartile 1 | Quartile 2 | | Quartile 3 | | Quartile 4 | |
| --- | --- | --- | --- | --- | --- | --- | --- | --- | --- | --- |
|  |  | β(95% CI) | p-value |  | β(95% CI) | p-value | β(95% CI) | p-value | β(95% CI) | p-value |
| Female | TgAb (IU/mL) | 0.17  ( -0.28, 0.62) | 0.43 | ref | 2.52  ( -7.23,12.27) | 0.59 | 11.44  ( -9.37,32.26) | 0.26 | -11.22  (-27.50, 5.05) | 0.16 |
|  | Free T3 (pg/mL) | 0  ( 0.00,0.01) | 0.27 | ref | 0.02  (-0.07, 0.11) | 0.63 | 0.1  (-0.08, 0.28) | 0.26 | 0.04  (-0.09, 0.17) | 0.50 |
|  | Free T4 (pmol/L) | 0  (-0.02,0.01) | 0.49 | ref | -0.44  (-0.84,-0.04) | 0.03 | -0.29  (-0.66, 0.08) | 0.12 | -0.22  (-0.68, 0.23) | 0.32 |
|  | Tg (ug/L) | 0.07  ( -0.12, 0.26) | 0.44 | ref | 4.91  ( -0.44,10.26) | 0.07 | 3.13  ( -3.76,10.01) | 0.35 | 2.99  ( -2.37, 8.36) | 0.25 |
|  | TSH (mIU/L) | -0.02  (-0.06, 0.03) | 0.49 | ref | -0.93  (-2.45, 0.58) | 0.21 | -0.1  (-2.06, 1.86) | 0.92 | -1.09  (-3.20, 1.02) | 0.29 |
|  | Total T4 (μg/dL) | -0.01  (-0.03, 0.02) | 0.69 | ref | -0.16  (-0.90, 0.58) | 0.64 | -0.19  (-0.70, 0.31) | 0.43 | -0.28  (-0.78, 0.21) | 0.24 |
|  | Total T3 (ng/dL) | -0.06  ( -0.29, 0.18) | 0.62 | ref | 3.23  ( -6.12,12.58) | 0.47 | 1.15  ( -5.08, 7.38) | 0.70 | -0.88  ( -7.24, 5.48) | 0.77 |
|  | TPOAb (IU/mL) | 0.65  ( -0.09, 1.39) | 0.08 | ref | 12.54  (-13.59,38.68) | 0.32 | 15.39  ( -7.08,37.86) | 0.17 | 12.51  (-10.32,35.34) | 0.26 |
| Male | TgAb (IU/mL) | 0.07  ( -0.10, 0.25) | 0.38 | ref | -6.03  (-15.01, 2.95) | 0.17 | -4.93  (-14.99, 5.13) | 0.31 | 0.43  (-13.98,14.84) | 0.95 |
|  | Free T3 (pg/mL) | 0  ( 0.00, 0.00) | 0.87 | ref | 0.01  (-0.08, 0.10) | 0.81 | 0.08  (-0.02, 0.17) | 0.11 | 0.02  (-0.11, 0.16) | 0.71 |
|  | Free T4 (pmol/L) | -0.01  (-0.03, 0.01) | 0.39 | ref | -0.45  (-1.13, 0.23) | 0.18 | -0.65  (-1.22,-0.07) | 0.03 | -0.17  (-0.59, 0.26) | 0.42 |
|  | Tg (ug/L) | -0.05  (-0.16, 0.06) | 0.33 | ref | -3  (-6.18, 0.18) | 0.06 | -0.98  (-4.37, 2.42) | 0.55 | -3.47  (-6.28,-0.65) | 0.02 |
|  | TSH (mIU/L) | 0  (-0.01,0.01) | 0.61 | ref | 0.04  (-0.27,0.35) | 0.79 | 0.15  (-0.05,0.34) | 0.13 | 0.1  (-0.25,0.46) | 0.56 |
|  | Total T4 (μg/dL) | 0  (-0.01,0.01) | 0.72 | ref | -0.15  (-0.49,0.19) | 0.37 | -0.2  (-0.59,0.20) | 0.31 | -0.19  (-0.52,0.14) | 0.24 |
|  | Total T3 (ng/dL) | 0.01  ( -0.23, 0.25) | 0.93 | ref | 1.67  ( -2.86, 6.20) | 0.45 | 3.35  ( -2.57, 9.27) | 0.25 | 0.58  ( -6.73, 7.89) | 0.87 |
|  | TPOAb (IU/mL) | -0.11  ( -0.64, 0.42) | 0.67 | ref | -3.84  (-13.41, 5.73) | 0.41 | 4.81  ( -8.04,17.66) | 0.44 | -10.66  (-21.39, 0.08) | 0.05 |

In the subgroup analysis, all covariates except gender were adjusted.
