# Supplementary material for: Association between the oxidative balance score and thyroid function: Results from the NHANES 2007–2012 and Mendelian randomization study
Source: PLoS One. 2024 Mar 18;19(3):e0298860. doi: 10.1371/journal.pone.0298860 (PMC10947682; doi:10.1371/journal.pone.0298860)
Supplement: S3 Table — (DOCX) [file pone.0298860.s004.docx]

**S3 Table. The table of the relationship between OBS and thyroid function within UIC subgroups.**

| Iodine intakes | Variable | Continuous | | Quartile 1 | Quartile 2 | | Quartile 3 | | Quartile 4 | |
| --- | --- | --- | --- | --- | --- | --- | --- | --- | --- | --- |
|  |  | β(95% CI) | p-value |  | β(95% CI) | p-value | β(95% CI) | p-value | β(95% CI) | p-value |
| Iodine deficient | TgAb (IU/mL) | -0.11  ( -0.95, 0.73) | 0.83 | ref | -1.53  (-10.90, 7.84) | 0.73 | 4.49  (-13.88,22.86) | 0.61 | -2.32  (-11.38, 6.74) | 0.59 |
|  | Free T3 (pg/mL) | 0  (0.00, 0.01) | 0.72 | ref | 0.02  (-0.09, 0.13) | 0.68 | 0.07  (-0.07, 0.21) | 0.31 | -0.03  (-0.17, 0.12) | 0.71 |
|  | Free T4 (pmol/L) | 0.01  (-0.01,0.03) | 0.46 | ref | 0  (-0.45,0.46) | 0.99 | 0.12  (-0.43,0.67) | 0.66 | 0.42  (-0.33,1.17) | 0.25 |
|  | Tg (ug/L) | 0.08  (-0.12, 0.28) | 0.56 | ref | -3.6  (-9.10, 1.90) | 0.18 | 3.34  (-2.48, 9.17) | 0.24 | 2.76  ( -8.71,14.23) | 0.62 |
|  | TSH (mIU/L) | -0.02  (-0.07,0.02) | 0.48 | ref | -1.19  (-3.30,0.91) | 0.25 | -1.16  (-3.56,1.25) | 0.32 | -1.25  (-3.82,1.32) | 0.32 |
|  | Total T4 (μg/dL) | 0.01  (-0.02, 0.04) | 0.73 | ref | -0.03  (-0.75, 0.70) | 0.94 | 0.18  (-0.53, 0.90) | 0.59 | 0.04  (-0.59, 0.67) | 0.88 |
|  | Total T3 (ng/dL) | -0.07  ( -0.28, 0.15) | 0.63 | ref | 1.15  ( -5.36, 7.65) | 0.71 | 3.77  ( -5.22,12.76) | 0.39 | -4.69  (-12.25, 2.88) | 0.21 |
|  | TPOAb (IU/mL) | -0.11  ( -0.95, 0.73) | 0.83 | ref | -6.98  (-29.10,15.13) | 0.51 | -5.3  (-26.12,15.52) | 0.60 | 2.6  (-26.32,31.52) | 0.85 |
| normal | TgAb (IU/mL) | 0.38  ( -0.02, 0.77) | 0.29 | ref | 2.71  (-10.55,15.98) | 0.67 | 9.54  (-6.95,26.04) | 0.24 | -5.32  (-23.09,12.45) | 0.53 |
|  | Free T3 (pg/mL) | 0  (0.00, 0.01) | 0.39 | ref | 0.01  (-0.04, 0.06) | 0.67 | 0.12  (-0.02, 0.26) | 0.10 | 0.08  (-0.02, 0.19) | 0.11 |
|  | Free T4 (pmol/L) | -0.02  (-0.03,0.00) | 0.31 | ref | -0.43  (-0.98, 0.12) | 0.12 | -0.71  (-1.12, -0.30) | **0.002** | -0.45  (-1.03, 0.12) | 0.11 |
|  | Tg (ug/L) | 0.04  (-0.08, 0.16) | 0.64 | ref | 4.18  (-0.89, 9.24) | 0.10 | 1.7  (-1.64, 5.04) | 0.30 | -1.01  (-4.68, 2.66) | 0.57 |
|  | TSH (mIU/L) | 0.01  (0.00,0.03) | 0.27 | ref | -0.15  (-0.64,0.35) | 0.54 | 0.84  (-0.34,2.01) | 0.15 | -0.26  (-1.71,1.18) | 0.70 |
|  | Total T4 (μg/dL) | -0.01  (-0.02, 0.00) | 0.37 | ref | -0.01  (-0.40, 0.38) | 0.97 | -0.32  (-0.70, 0.05) | 0.09 | -0.47  (-0.98, 0.03) | 0.07 |
|  | Total T3 (ng/dL) | -0.03  ( -0.19, 0.14) | 0.80 | ref | 2.28  ( -3.81, 8.37) | 0.44 | 0.68  ( -3.79, 5.15) | 0.75 | 1.4  ( -4.75, 7.56) | 0.63 |
|  | TPOAb (IU/mL) | 0.38  ( -0.02, 0.77) | 0.29 | ref | 10.91  ( -5.93,27.74) | 0.19 | 14.2  (0.26,28.13) | **0.05** | 1.08  (-14.59,16.76) | 0.89 |
| excessive iodine intake | TgAb (IU/mL) | 0.89  ( -0.86, 2.64) | 0.47 | ref | -9.61  (-40.02,20.81) | 0.51 | -13.58  (-38.99,11.83) | 0.27 | -12.26  (-33.52, 8.99) | 0.24 |
|  | Free T3 (pg/mL) | 0  (-0.01, 0.01) | 0.77 | ref | 0.02  (-0.14, 0.17) | 0.83 | -0.04  (-0.19, 0.11) | 0.58 | 0  (-0.22, 0.22) | 0.99 |
|  | Free T4 (pmol/L) | -0.01  (-0.05,0.02) | 0.61 | ref | -1.12  (-2.69,0.44) | 0.15 | -0.64  (-2.23,0.96) | 0.41 | -1.12  (-2.45,0.21) | 0.09 |
|  | Tg (ug/L) | -0.15  (-0.44, 0.14) | 0.46 | ref | 1.61  ( -7.56,10.78) | 0.71 | -5.62  (-19.10, 7.87) | 0.39 | -4.14  (-11.76, 3.48) | 0.27 |
|  | TSH (mIU/L) | -0.01  (-0.04,0.01) | 0.41 | ref | 0.13  (-0.54,0.80) | 0.68 | -0.15  (-0.79,0.48) | 0.61 | -0.04  (-0.81,0.74) | 0.92 |
|  | Total T4 (μg/dL) | -0.01  (-0.04,0.02) | 0.59 | ref | -0.76  (-1.66,0.13) | 0.09 | -0.57  (-1.39,0.25) | 0.16 | -0.51  (-1.33,0.32) | 0.21 |
|  | Total T3 (ng/dL) | 0.06  ( -0.37, 0.48) | 0.82 | ref | 4.81  ( -5.14,14.76) | 0.32 | 2.86  ( -5.45,11.17) | 0.48 | 6.38  ( -4.21,16.97) | 0.22 |
|  | TPOAb (IU/mL) | 0.89  ( -0.86, 2.64) | 0.47 | ref | 21.23  (-13.24,55.69) | 0.21 | 39.22  ( -27.47,105.91) | 0.23 | 11.79  (-43.65,67.24) | 0.66 |

In the subgroup analysis, all covariates except UIC were adjusted.
